# Timing of exposure impacts how organophosphorus pesticides affect the developing brain in the planarian *Dugesia japonica*

**DOI:** 10.1007/s00204-026-04415-x

**Published:** 2026-05-13

**Authors:** Rebecca T. Somach, Danielle Ireland, Hao P. Tran, Gustav Allotey, Eva-Maria S. Collins

**Affiliations:** 1 Present Address: Biology Department, Swarthmore College, Swarthmore, PA USA; 2 Department of Physics and Astronomy, Swarthmore College, Swarthmore, PA USA; 3 Department of Neuroscience, Perelman School of Medicine, University of Pennsylvania, Philadelphia, PA USA; 4 Center for Excellence in Environmental Toxicology, University of Pennsylvania, Philadelphia, PA USA

**Keywords:** Profenofos, Chlorpyrifos, Malathion, Diazinon, Dichlorvos, Window of vulnerability

## Abstract

Exposure to organophosphorus pesticides (OPs) during pregnancy has been suggested to cause adverse outcomes on neural development of the fetus but the mechanisms underlying developmental neurotoxicity of OPs remain unclear. While OPs share acetylcholinesterase (AChE) as the primary molecular target, different OPs have been shown to induce different effects in laboratory studies. Previously, we found that OPs differentially affect regenerating planarians when applied on the first day of regeneration. To determine if the timing of OP exposure affects adverse outcomes, here, we performed a comparative study of 5 OPs—chlorpyrifos (17.8 μM), diazinon (16.2 μM), dichlorvos (1 μM), profenofos (10 μM), and malathion (56.2 μM)—in the planarian *Dugesia japonica*. We evaluated how effects on behavior and nervous system morphology changed with OP exposure starting on different days of regeneration/development. We found that some toxic effects were dependent on the developmental stage during exposure. For example, profenofos impaired brain development when exposure started on day 1 of development but caused lethality when exposure started during later stages. Inhibition of the enzymatic activity of AChE on day 12 of exposure was not affected by the different exposure paradigms, suggesting that the differential effects were either due to effects on the non-enzymatic functions of AChE or on other targets during neurodevelopment. This work provides insight into which neurodevelopmental key events are affected by OP exposure, enabling future mechanistic work to identify the targets underlying OP developmental neurotoxicity.

**Supplementary Information:**

The online version contains supplementary material available at 10.1007/s00204-026-04415-x.

## Introduction

Organophosphorus pesticides (OPs) are widely used in agriculture throughout the world (Mora-Gutiérrez et al. 2021). OPs are effective against many pests because they inhibit acetylcholinesterase (AChE) (Mileson 1998; Robb et al. 2025), a key enzyme for regulating neuromuscular communication in many animals, including humans. Chronic exposure to OPs at low levels that do not cause significant AChE inhibition in humans during development has been associated with neurodevelopmental issues, such as effects on attention, memory, cognition, and motor function (Eaton et al. 2008; Muñoz-Quezada et al. 2013; González-Alzaga et al. 2014; Burke et al. 2017; Sapbamrer and Hongsibsong 2019; Richardson et al. 2019; Todd et al. 2020). As first recognized by a National Research Council Report in 1993 (National Research Council (U.S.) 1993), children may be uniquely susceptible to OP neurotoxicity due to increased exposure, differences in uptake and metabolism, and the highly dynamic nature of nervous system development involving numerous critical key events, whose disruption can lead to long lasting adverse effects (Faustman et al. 2000; Rice and Barone 2000; Heyer and Meredith 2017). These periods of important developmental events are termed critical periods and include important processes such as proliferation, migration, and synaptogenesis (National Research Council (U.S.) 1993; Rodier 1994; Rice and Barone 2000; Selevan et al. 2000). Thus, disruption of specific key events can lead to chemical-specific “windows of vulnerability” or developmental periods during which chemical exposure is the most detrimental and can lead to permanent adverse effects in the adult organism. Because different key events are driven by different molecules and signaling pathways, identifying OP-specific windows of vulnerability can provide insight into the molecular targets of that OP during neurodevelopment.

Laboratory studies have shown causal relationships between OP exposure during neurodevelopment and adverse outcomes on the molecular, cellular, and behavioral levels that cannot be explained by AChE inhibition alone (reviewed in (Burke et al. 2017; Carr et al. 2018; Costa 2018)). Although some OPs, particularly chlorpyrifos (CPF), have been shown to have effects on many secondary targets, such as mitochondria, cytoskeletal proteins, glial cells, various neurotransmitter receptors, and other non-AChE esterases (Pope 1999; Aldridge et al. 2003; Garcia et al. 2005; Burke et al. 2017; Hernandez-Toledano and Vega 2021; Hernández et al. 2025), how these targets contribute to OP developmental neurotoxicity is still unclear and under intense investigation. For example, disrupted neurite outgrowth is a commonly observed phenotype associated with developmental exposures to OPs (Hernández et al. 2025). In developing zebrafish, exposure to CPF oxon disrupted axonal growth at concentrations that also disrupted touch-induced swimming behavior, suggesting these structural defects have functional relevancy (Yang et al. 2011). Effects on neurite outgrowth may be explained by observations that some OPs, or their active oxon forms, directly interact with cytoskeletal proteins both in vitro and in vivo (reviewed in (Flaskos 2014; Hernandez-Toledano and Vega 2021)). Additionally, multiple targets may play a role depending on the timing of exposure as studies in rats have suggested that CPF first primarily targets neurons during early prenatal neurodevelopment and then glia during later stages (Garcia et al. 2005).

Studying potential windows of vulnerability of OPs during critical periods of neurodevelopment is challenging in mammalian models because assessing multiple developmental cohorts simultaneously is extremely time and resource intensive (Meigs et al. 2018). A few studies have made comparisons of exposure timing for CPF but have thus far relied on comparisons across multiple studies (Qiao et al. 2002; Icenogle et al. 2004; Garcia et al. 2005). Additionally, different OPs do not produce the same adverse outcomes (Pope 1999; Slotkin et al. 2006; Richendrfer and Creton 2015; Ireland et al. 2022b), underscoring the need for comparative studies that examine multiple OPs in parallel to reveal both shared and distinct mechanisms. Thus, because of the financial, temporal, and ethical challenges associated with mammalian studies, there is a significant data gap in our understanding of the mechanisms underlying OP developmental neurotoxicity, which is crucial for implementing protective regulatory guidelines. New Approach Methods (NAMs) amenable to cost-effective, rapid screening, including human and rat primary neuron cell culture models (Smirnova et al. 2014; Carstens et al. 2022) and small organismal models (nematodes, developing zebrafish, planarians; reviewed in (Collins et al. 2024)) overcome the limitations of mammalian testing.

Studies with human cell culture models are particularly well suited for mechanistic studies, allowing for evaluation of direct target binding, effects on a wide range of cellular processes, such as apoptosis, mitochondrial function, autophagy, and on neuronal morphology and function (Hernández et al. 2025). However, many OPs require desulfuration by cytochrome P450s into their bioactive metabolites (oxon) (Amitai et al. 1998), which is often not fully functional in in vitro systems, confounding results or relying on exposure directly to the oxon. Recently, the use of human induced pluripotent stem cells derived neuron/astrocyte co-cultures have been developed with metabolic competency (Di Consiglio et al. 2020). The use of small organismal models can further bridge the gap between in vitro and mammalian studies by allowing for evaluation of systems effects in an intact organism. For example, evaluation of lethality, hatching rate, morphology, mitochondrial bioenergetics, and photomotor behavior in developing zebrafish revealed greater toxicity across endpoints with CPF than diazinon (DZN) (Cao et al. 2018). To fully understand how OPs disrupt the developing nervous system and how this leads to functional changes in adult organisms, data will need to be leveraged from multiple complementary NAMs.

**Fig. 1:**
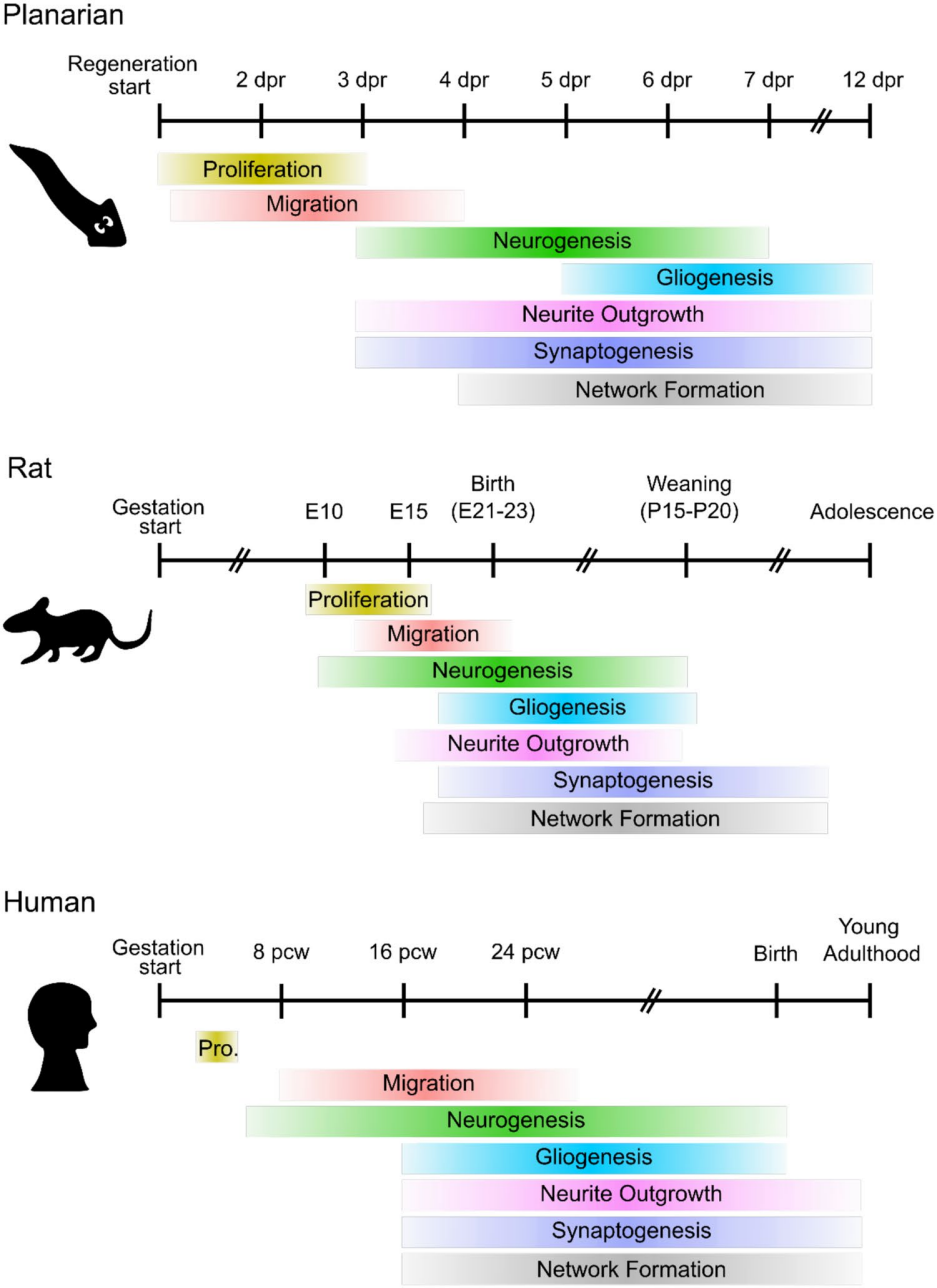
Planarians share key neurodevelopmental events with rats and humans. Comparison of the timing of major neurodevelopmental key events (defined in Supplementary Table 1) in planarians (Inoue et al. 2004; Nishimura et al. 2007; Cebrià 2007; Wenemoser and Reddien 2010; Lapan and Reddien 2011; Ross et al. 2017; Ireland and Collins 2023; Chandra et al. 2023), rats (König et al. 1975; Das 1979; Altman and Bayer 1980; Bayer and Altman 1990; De Carlos and O’Leary 1992; Frassoni et al. 1995; Molnár et al. 1998; Rice and Barone 2000; Gressens 2000; Sauvageot 2002; Miller and Gauthier 2007; Semple et al. 2013; Hayashi et al. 2015; Ma et al. 2018; Zeiss 2021; Lattke and Guillemot 2022), and humans (Semple et al. 2013; Budday et al. 2015; Courchesne et al. 2019; Zeiss 2021; Zhou et al. 2024). The planarian timeline is represented in days post regeneration (dpr). The rat timeline is presented in either embryonic day (E) or postnatal day (P). The human timeline is presented in terms of post-conception week (pcw)

Freshwater planarians have emerged as a well-suited small invertebrate organismal model for studying OP developmental neurotoxicity (Ireland and Collins 2023). The planarian nervous system consists of about 2000–10,000 neurons (Brown et al. 2018) in which the central nervous system is organized into two ventral nerve cords and a central ganglia (brain-like structure) (Ross et al. 2017). Nearly all planarian neuronal genes (~ 95%) have human homologs (Mineta et al. 2003). Some species of planarians, including *Dugesia japonica,* reproduce asexually by binary fission and the tail offspring grows all anterior structures, including a new brain over the course of 2 weeks (Cebrià 2007). Critically, although the complexity of the nervous systems differs dramatically, key neurodevelopmental events, such as migration, neurogenesis, gliogenesis, neurite outgrowth, and synaptogenesis, are conserved between planarians and mammals (Fig. 1; Rice and Barone 2000; Cebrià 2007; Hartenstein and Stollewerk 2015; Ross et al. 2017; Zeiss 2021; Ireland and Collins 2023). Moreover, while the absolute timing of events differs significantly due to the different time scales of neurodevelopment across species, the relative order of events is similar. For example, neurons and glia develop in the same order, with neurogenesis preceding gliogenesis (Qian et al. 2000; Chandra et al. 2023).

Taken together, these qualities make planarians a good model of nervous system development. Additionally, planarians have unique benefits for OP neurotoxicity studies, which we have recently reviewed (Ireland and Collins 2023): *D. japonica* have the ability to bioactivate OPs at all life stages (Ireland et al. 2022a) and have cholinesterases that share similarities with human cholinesterases, can be inhibited by OPs, and can be reactivated by oximes (Hagstrom et al. 2017, 2018).

Previously, we showed that adult and regenerating planarians have distinctive behavioral phenotypes when continuously exposed for 12 days to 7 different OPs (acephate, CPF, DZN, dichlorvos (DDVP), malathion (MT), parathion and profenofos (PFS)) and that the outcomes did not correlate with the level of AChE inhibition on day 12 of exposure (Ireland et al. 2022b). Through comparison of toxicological profiles with mechanistic control compounds known to affect suggested targets of OPs, we found that certain behavioral readouts associated with cholinergic toxicity while others did not. Together, these results suggested that these OPs have different targets in planarians aside from their shared target of AChE, similar to previous results in mammals (Pope 1999; Slotkin et al. 2006). In the current study, we compared outcomes when OP exposure occurred on different days of development for the 5 OPs that showed sublethal behavioral effects (CPF, DZN, DDVP, MT, and PFS). To identify critical windows of vulnerability wherein exposure must occur to induce adverse outcomes, we exposed planarians to these 5 OPs at different stages of development and measured effects on behavioral outcomes and brain morphology.

## Materials and methods

### Planarian culture

Asexual freshwater planarians of the species *D. japonica* that have been cultured in the lab for over a decade were used for all experiments. Planarians were maintained in planarian water (Ireland et al. 2025a) in plastic containers (Ziploc, Bay City, MI, and ACME SignatureSelect, Malvern, PA) at 20 °C in a refrigerated incubator (Panasonic, Newark, NJ) in the dark. Planarians were fed organic beef liver 1–2 × /week (obtained from a local farm) and cleaned 2–3 × /week. Planarians were fasted for at least 5 days before experiments.

### Chemical preparation

The 5 OPs and their tested concentrations are listed in Table 1. In our previous work (Ireland et al. 2022b), we examined a range of concentrations and determined the benchmark concentrations that caused behavioral effects for each OP. Here, we chose a single concentration for each OP that showed robust sublethal behavioral effects without systemic toxicity in our previous work (Ireland et al. 2022b). All OPs were diluted in dimethyl sulfoxide (DMSO) (Fisher Bioreagents, Pittsburgh, PA, CAS: 67-68-5) so that the final DMSO concentration was 0.5% (v/v). For the solvent control, planarians were exposed to 0.5% (v/v) DMSO, which we have previously shown to not affect *D. japonica* behavior or neuronal morphology (Hagstrom et al. 2015; Ireland et al. 2020). OP purity was independently confirmed by high performance liquid chromatography/mass spectrometry with a UV detector (Lotus Separations, Princeton, NJ) (Supplementary File 1).

**Table 1:** Chemical overview

Chemical name	CAS	Supplier	Concentration (μM)	Vendor purity (%)	Verified purity (%)
Chlorpyrifos (CPF)	2921-88-2	Sigma Aldrich	17.8	99.8	99.50
Diazinon (DZN)	333-41-5	Sigma Aldrich	16.2	99.6	99.50
Dichlorvos (DDVP)	62-73-7	Sigma Aldrich	1	99.4	> 99.9
Malathion (MT)	121-75-5	Sigma Aldrich	56.2	99.5	> 99.9
Profenofos (PFS)	41198-08-07	Chem Service	10	96.2	98.30

### Planarian regeneration protocol and chemical incubation

**Fig. 2:**
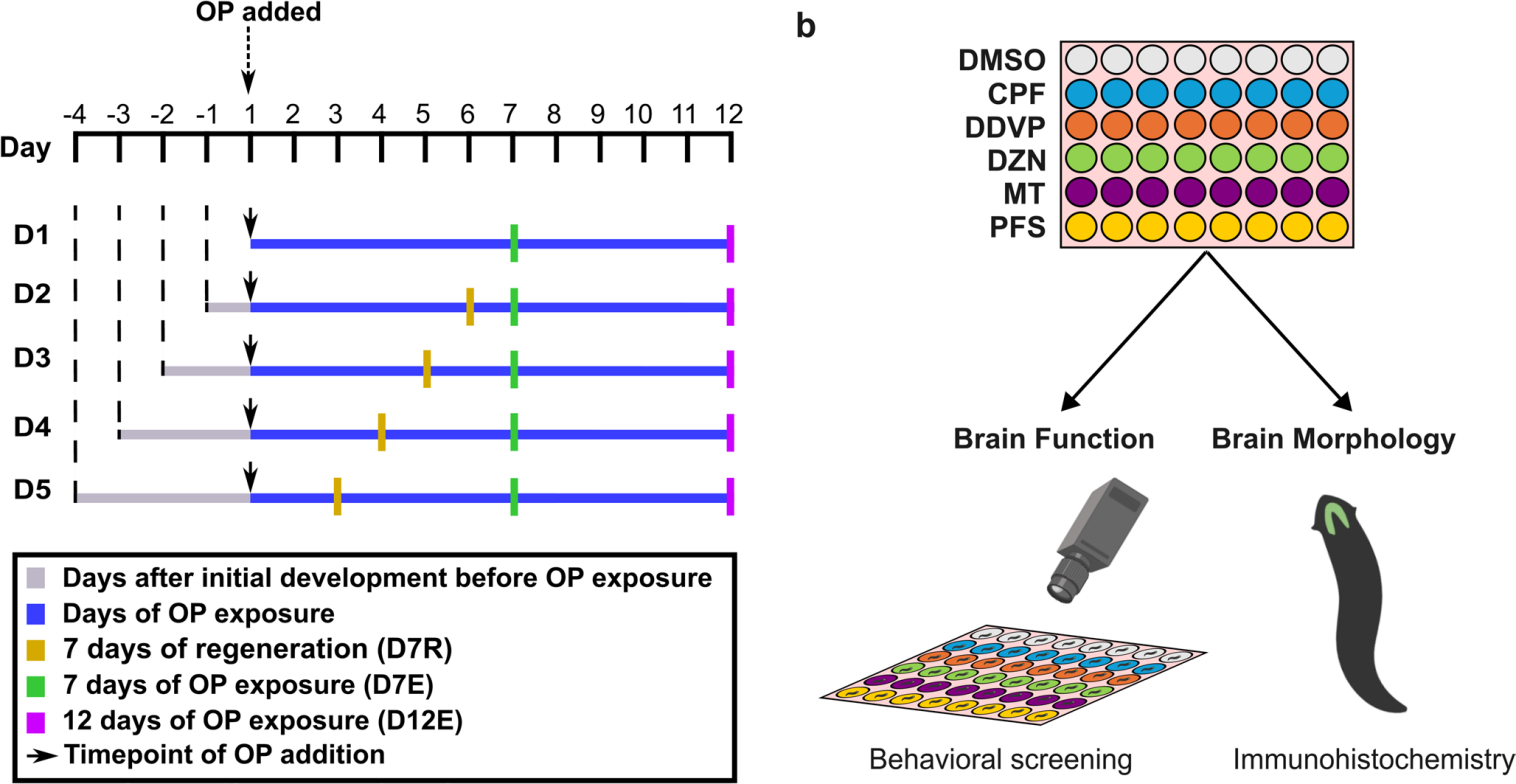
Overview of experimental timeline and workflow. (**a**) Timeline for each developmental group assigned by development day when the OP was added (D1-D5). OPs were added on Day 1 for each experimental group. For the D1 exposure group, day 7 after the start of development and day 7 of OP addition coincide and is depicted with a green vertical line. Exposure was for a maximum of 12 days after OP addition. (**b**) Schematic of plate setup and experimental workflow. Partially created in BioRender. Somach, R. (2026) BioRender.com/7tl0bmx

Intact planarians were transected pre-pharyngeally with a 70% ethanol sterilized razor blade (VWR, Radnor, PA) at either 4, 3, 2, or 1 day prior to OP incubation or on the same day as the start of OP incubation (“Development Day when OP was applied”; hereafter referred to as developmental groups D5-D1, see Fig. 2). The tail pieces were placed in 48-well plates (tissue culture treated 25-108MP, Genesee Scientific, El Cajon, CA) with one specimen per well in 200 µl planarian water. On the day of chemical addition, 100 µL of planarian water was removed and 100 µL of the respective 2 × OP stock was added to each well. The plates were sealed with sealing film (ThermalSeal RTS, Excel Scientific, Victorville, CA) to prevent evaporation. Exposure was static for 12 days, with no solution exchanges. During exposure, the plates were stored in a Panasonic incubator at 20  °C continuously in the dark. Planarians were assessed for behavior and brain morphology at three time points: 7 days after regeneration (D7R), 7 days after OP exposure (D7E) or 12 days after OP exposure (D12E). Planarians at D7R provided a fixed developmental time point of comparison but had varying durations of OP exposure across the developmental groups. Thus, D7E was also measured to ensure that any effects not present at D7R were not a result of shorter OP exposure times. After 12 days of regeneration, planarians are considered ‘adults’ (Zhang et al. 2019) and thus would be at the same developmental stage from day 12 of regenerating onward, so only D12E was evaluated to keep exposure duration consistent.

### Planarian behavior

For behavior experiments, developmental groups D1, D2, D3, D4, and D5 were examined. Screening was performed on an automated platform, described in detail in our previous work (Zhang et al. 2019; Ireland et al. 2020, 2022b; Bayingana et al. 2023; Fuselier et al. 2023). For each developmental group, one 48-well plate consisting of one row of 8 worms per OP was tested per experiment. Behavior experiments were performed in triplicate for a total of n = 24 planarians per OP and developmental group. The orientation of the OP location in the plate was shifted down 2 rows in each replicate to control for edge effects (Zhang et al. 2019). Measured behavioral endpoints are detailed in Table 2. Behavioral endpoints were measured in the same manner as in our previous work (Ireland et al. 2022b; Fuselier et al. 2023) using custom scripts in MATLAB (MATLAB R2024b, MathWorks, Natick, MA).

**Table 2:** Behavior endpoints. Post hoc adjustment includes the Benjamini and Hochberg (BH) method for correction for multiple testing (Benjamini and Hochberg 1995)

Endpoint	Description	Statistical test	Tails
Lethality	% dead	Fisher’s exact (BH correction)	Greater
Stickiness	% stuck individuals	Fisher’s exact (BH correction)	Greater
Scrunching	% scrunching planarians	Fisher’s exact (BH correction)	Less
Phototaxis	% phototaxis individuals	Fisher’s exact (BH correction)	Less
Speed light	Mean speed in blue light period	Welch’s ANOVA, Post hoc Tamhane-Dunn	Both sides
Speed dark	Mean speed in dark period	Welch’s ANOVA, Post hoc Tamhane-Dunn	Both sides
Resting light	Fraction of time spent resting during blue light period	Kruskal Wallis, Post hoc Dunn (BH correction)	Both sides
Resting dark	Fraction of time spent resting during dark period	Kruskal Wallis, Post hoc Dunn (BH correction)	Both sides
Anxiety	Fraction of time spent in outer region of well	Kruskal Wallis, Post hoc Dunn (BH correction)	Less
Noxious stimuli strength (NS Strength)	Median displacement at the end of noxious heat	Welch’s ANOVA, Post hoc Tamhane-Dunn	Both sides
Locomotor bursts	Sum of locomotor bursts during the phototaxis assay	Negative binomial GLM, Post hoc Dunnett (BH correction)	Both sides

Statistical analysis was performed in R (Version 4.4.3, (R Core Team 2016)) as previously described (Fuselier et al. 2023) (Table 2). For all statistical tests, comparisons were made with the respective in-plate 0.5% (v/v) DMSO controls and adjusted p-values < 0.05 were considered significant. To decrease false positives due to plate-to-plate variability, we calculated “biological relevancy cutoffs” (Zhang et al. 2019) as the 5th − 95th percentiles of the compiled scores of the controls for each readout. To this end, we screened an additional three plates of 0.5% (v/v) DMSO exposed controls that were amputated, exposed, and screened in the same paradigm as our OP exposed planarians making 15 DMSO control groups. Thus, in total we had 25 control populations each with n = 24. Statistically significant hits that fell within the biological relevancy cutoffs were excluded. Graphs were produced in R with some code to generate graphs written with assistance from ChatGPT and Google Gemini. The authors reviewed and edited the code as needed and take full responsibility for the content of the publication. The compiled behavioral data and associated p-values are provided in Supplementary File 1.

### Immunohistochemistry

Immunohistochemistry (IHC) was performed as previously described (Hagstrom et al. 2015), with minor modifications. Following OP exposure, planarians were washed 3 times in planarian water in the 48-well plate and transferred to a 1.7 mL microcentrifuge tube (VWR) using a P200 pipettor (Corning, Corning, NY) with a cut-off P200 tip (USA Scientific, Ocala, FL) with approximately 8 planarians per tube. The water was removed and replaced with 4% paraformaldehyde (v/v, Ted Pella, Redding, CA, CAS: 30525-89-4) for fixation for 15 min at room temperature. Bleaching in 6% (v/v) hydrogen peroxide (Sigma-Aldrich, CAS:7722-84-1) in methanol (Sigma-Aldrich, St. Louis, MO, CAS: 67-56-1) occurred overnight at room temperature on a nutator under a bright light. Blocking occurred overnight at 4 °C in antibody blocking buffer (10% goat serum (ThermoFisher Scientific, Waltham, MA) in phosphate buffered saline (PBS; MP Biomedicals, Irvine, CA) with 0.3% TritonX-100 (Sigma Aldrich, CAS: 9036-19-5)). We used two primary antibodies to assess neurons and glia cells, respectively. The primary antibody for labeling neurons was a mouse anti-synapsin antibody (3C11 (anti SYNORF1), deposited to the Developmental Studies Hybridoma Bank by Buchner, E. (DSHB Hybridoma Product 3C11 (anti SYNORF1))). The anti-synapsin antibody was diluted 1:100 in antibody blocking buffer. To label glia, we used a mouse anti-Glial Fibrillary Acidic Protein (GFAP) antibody (Developmental Studies Hybridoma Bank, N206A/8, anti-GFAP, deposited to the DSHB by Trimmer, J.S.), which was diluted 1:200 in antibody blocking buffer overnight at 4 °C. After primary antibody staining, the samples were washed with 0.1% Tween-20 (Sigma-Aldrich, CAS: 9005-64-5) and 0.3% TritonX-100 in PBS (PBSTT) 4 times for 20–30 min each at room temperature on a nutator. Samples were then incubated overnight on a nutator at 4 °C with Goat anti-Mouse IgG (H + L) Cross-Adsorbed Secondary Antibody, Alexa Fluor™ 546 (ThermoFisher Scientific- Invitrogen A-11003), diluted 1:1000 in antibody blocking solution. Planarians were washed 4 times for 20–30 min per wash on a nutator at room temperature in the dark with PBSTT. The samples were washed in 50% glycerol (OmniPur, CAS: 56-81-5) and 50% PBSTT and then placed in 100% glycerol. Planarians were mounted on tunnel slides (Hackler et al. 2025). The slides were imaged on an inverted IX81 spinning disc confocal microscope (Olympus DSU) using an ORCA-ER camera (Hamamatsu Photonics) and imaged with Slidebook software (2023, Intelligent Imaging Innovations, Inc.). Planarians from developmental groups D5, D3, and D1 were each examined at timepoints D7R, D7E, and D12E. Sixteen planarians total were stained for each antibody and OP per developmental group and timepoint. At least 5 samples per group (combination of OP, timepoint, developmental group, and antibody), across at least 2 independent exposure experiments, were analyzed for brain size.

To analyze the relative size of the brain, we quantified the ratio of the width of the brain over the width of the head (“brain-to-head width ratio”, Supplementary Fig. 1). Quantification was manually performed in FIJI (Schneider et al. 2012) by analyzing the maximum intensity projections of z-stacks taken with a 10 × objective. The measurements were taken independently by at least 2 researchers who did not know the identity of the images, thus removing bias. The measurement values were averaged between the researchers for the final values (Supplementary File 2). Brains that failed to regrow fully, defined by failure to develop neural tissue or failure to reconnect across the central ganglia (Supplementary Fig. 1), were given a measurement of 0. Measurement data were compiled and analyzed in Microsoft Excel and MATLAB. Statistical analysis was performed in R using the Kruskal–Wallis test with a post-hoc Dunn’s test. *P*-values < 0.05 were considered significant. Graphs were produced in R. To determine whether there was a statistically significant difference in the number of planarians without a properly reformed brain in PFS worms across the different developmental groups, we compared the proportion of brains that did or did not develop to in each group separated by antibody, developmental group, and timepoint when fixed against the respective DMSO exposed controls for each group, 100% of which had developed brains with different N’s per group (N between 7–15 per group) using a Fisher’s exact test. *P*-values < 0.05 were considered as significantly different.

### Ellman assays

After 12 days of OP exposure, 24 planarians were washed once with planarian water in the 48 well plate and then transferred to a 50 mL conical tube (Avantor, Randnor Township, PA) and washed two more times. They were then homogenized in 200 μL 1% (v/v) Triton X-100 in 1 × PBS as described in (Hagstrom et al. 2017, 2018; Fuselier et al. 2023). Protein concentration was determined by a Coomassie (Bradford) protein assay kit (Thermo Scientific) and samples within the same experiment were diluted to the same concentration. An Ellman assay (Ellman et al. 1961) was performed on the clarified homogenate using an AChE Activity Assay kit (Sigma-Aldrich) in 96-well plates (Genesee Scientific). Absorbance was read at 412 nm every minute for 10 min using a SpectraMax ABS Plus (Molecular Devices, San Jose, CA) spectrophotometer or Varioskan ALF Multimode Microplate Reader (ThermoFisher Scientific). AChE activity was calculated as the rate of change of absorbance per minute during the linear portion of the reaction and compared to 0.5% (v/v) DMSO control samples (set at 100% activity) tested on the same day. Each homogenate sample was run with 3 technical replicates in the 96-well plate. Two–three independent experiments (biological replicates) were performed and averaged. Planarians were examined at D1 and D5. A 2-way ANOVA was performed in R comparing amputation date. P-values < 0.05 were considered significant. Graphs were produced in R.

## Results

### Timing of exposure influences OP behavioral phenotypes

We have previously shown that sublethal exposure to either CPF, DDVP, DZN, MT, or PFS induces distinct behavioral phenotypes in regenerating planarians when exposure started on day 1 of regeneration/development (Ireland et al. 2022b). Here, we asked whether the timing of exposure would affect behavioral outcomes. We exposed regenerating planarians to the 5 OPs individually at developmental points separated by 24-h intervals across days 1–5 of regeneration (D1-D5, Fig. 2). By day 5, most neurodevelopmental key events have at least begun and the major structures of the planarian brain have formed, although full growth to an adult-like size and completion of network formation can take up to 12 days (Fig. 1) (Cebrià 2007; Hagstrom et al. 2015; Ross et al. 2017). Thus, these exposure windows allowed for evaluation across almost all neurodevelopmental key events, while separation by 24 h allowed for distinction of the exact mix of active key events. We evaluated planarian behavior at three time points: 7 days after planarian regeneration (D7R), 7 days after OP exposure (D7E)—to account for effects from the duration of exposure—and 12 days after OP exposure (D12E) (Fig. 2). The D7E and D12E timepoints of the D1 planarians are equivalent to the time points previously studied (Ireland et al. 2022b).

**Fig. 3:**
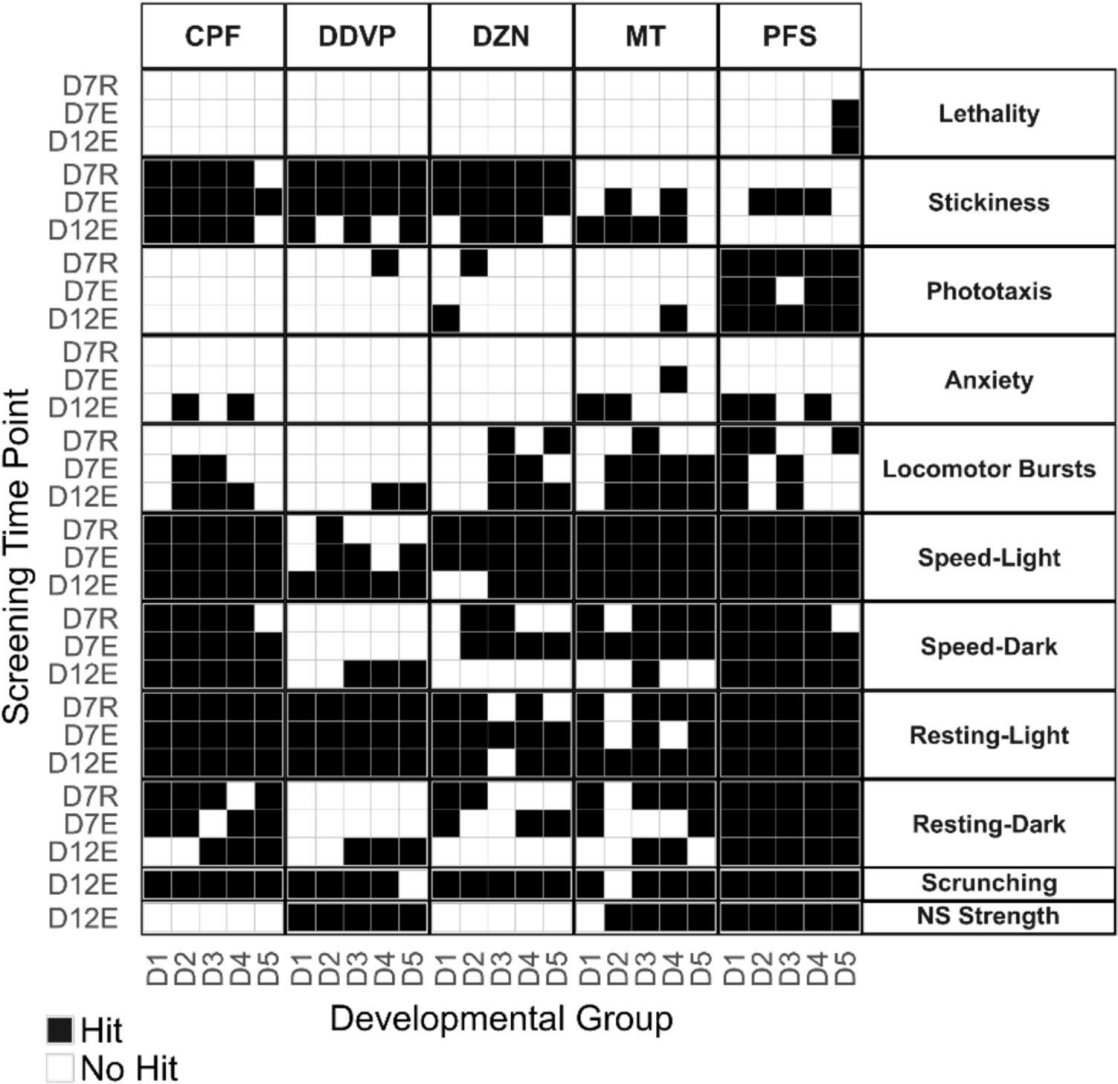
Results from behavioral screen of 5 OPs with exposure starting at different times. Each box represents whether the readout was significantly different (*p* < 0.05) than 0.5% DMSO exposed controls (see Methods). Significantly different readouts are marked in black, non-significantly different readouts are marked in white. For D1, D7R and D7E are the same timepoint—this point was reproduced for comparison. NS: noxious stimuli

Each of the 5 OPs caused a distinct behavioral profile (Fig. 3), in agreement with our previous results (Ireland et al. 2022b). Across all OPs, some readouts and time points did not differ across the developmental groups. However, some readouts showed differential effects dependent on the timing of exposure. For several OPs, certain readouts at D7R were present in all developmental groups except for D5, for example speed-dark in CPF-exposed planarians. As these readouts were often still affected in all developmental groups at D7E, this suggests that the reduced exposure time for developmental group D5 and D7R (only 2 days of exposure) was not sufficient to induce a behavioral effect. OP-specific trends were also observed.

For CPF, increased resting in the dark period at D12E was only observed in D3-D5 planarians but most of the other bioactive readouts, including speed-light, resting-light and scrunching, were not affected by the timing of exposure.

With DDVP exposure, we observed that on D12E, planarians exposed at D3-D5, but not earlier, showed increased resting in the dark and decreased speed in the dark. Similarly, effects on locomotor bursts at D12E were only observed in planarians in developmental groups D4 and D5. In contrast, scrunching was significantly decreased in groups D1-D4, but not in D5.

For DZN exposure, speed in the light period at D12E was only affected in planarians that were exposed at D3 or later. This was not true at the D7R or D7E time points where all developmental groups were affected. In contrast, phototaxis at D12E was only affected in the D1 developmental group but not in the more developed groups.

With MT exposure, several readouts showed differential effects based on the timing of exposure. The increased stickiness phenotype at D12E was present in all developmental groups except D5. Similarly, anxiety at D12E showed significant effects in the D1 and D2 groups, but not in the later exposure groups. In contrast, both the locomotor bursts at D7E and NS strength readouts only showed significant effects in developmental groups D2-D5, with effects missing from D1. Additionally, resting in both the dark and light at D7R showed significant changes in all other developmental groups aside from the D2 group.

PFS was the only OP that caused significant lethality, though only in the D5 group. We chose the concentrations in our current study because they did not cause lethality in regenerating planarians that were exposed immediately after amputation in our previous work (Ireland et al. 2022b). In agreement with this observation, the analogous D1 planarians did not show lethality here. Many behavioral readouts were also affected by PFS exposure, but were largely not affected by the timing of exposure.

### Timing of OP exposure affects brain size

**Fig. 4:**
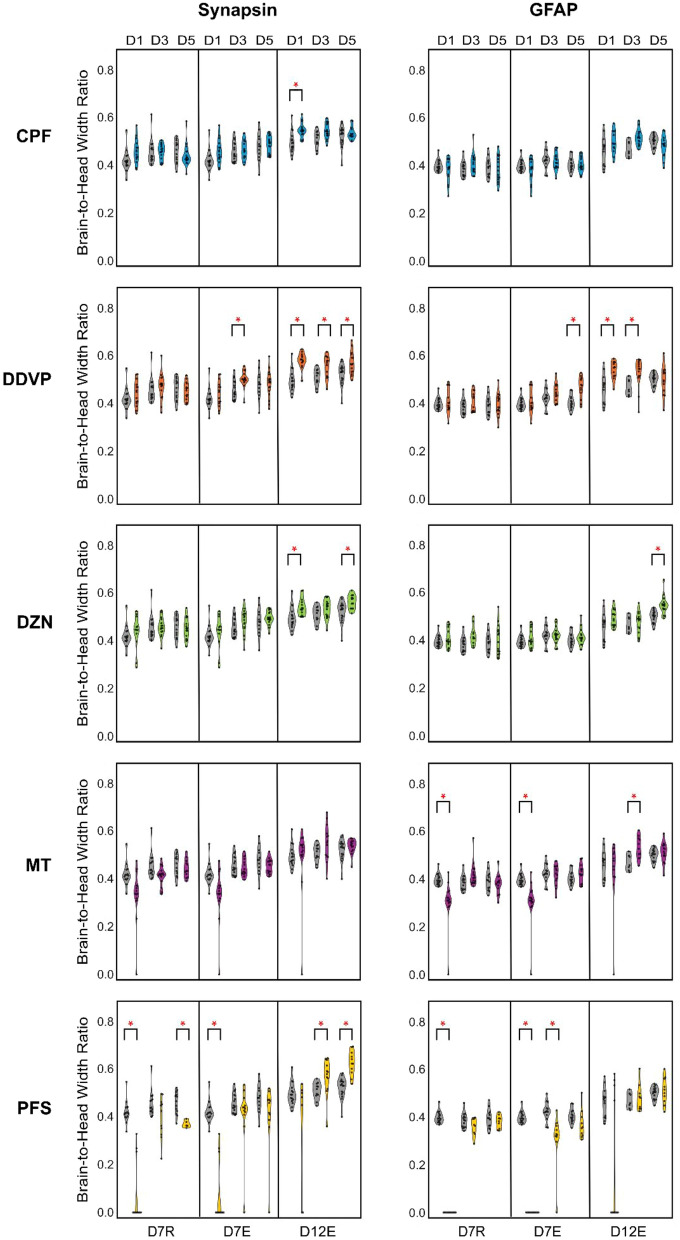
Measurements of relative brain size using synapsin and GFAP reveal dependence on developmental stage during exposure for effects**.** All measurements were made of the brain width to head width ratio (See methods and Supplementary Fig. 1). Gray violin plots represent DMSO exposed controls, colored violin plots represent OP exposed planarians. Significant results are marked with a * where *P* < 0.05 as measured by a Kruskal–Wallis test with a post-hoc Dunn’s test. Graphs were produced in R. N = 5–17 animals per group

Our behavioral results indicated that OP exposure causes functional changes in developing planarians, and that the nature of these effects depend on the developmental stage at the time of exposure. To investigate whether these functional impairments correlated with alterations in brain morphology, we exposed regenerating planarians from different developmental groups (D1, D3, D5) to the OPs and measured the brain-to-head width ratio, which we also refer to as relative brain size (Fig. 4). This measure was previously used to assess effects of neurotoxicants, including CPF and DDVP, on brain development (Hagstrom et al. 2015). We used IHC to visualize neurons with an antibody against synapsin (*Drosophila*) and glia using an antibody against GFAP (human). Due to the larger number of samples needed for these experiments (6 conditions X 3 time points X 2 antibodies = 36 samples per developmental group, per experiment) and the manual nature of the IHC experiments and associated imaging, we only evaluated developmental groups D1, D3 and D5 in these experiments to provide insights in the importance of exposure during early, mid, or late regeneration/development, respectively.

For CPF, DDVP, and DZN, we observed increases in relative brain size that were exposure window specific (Fig. 4). In CPF-exposed planarians, effects were only observed for synapsin-labeled brains at D12E in the D1 developmental group. In contrast to CPF, DDVP and DZN showed effects on both neurons and glia. DDVP exposure produced significant increases in size in synapsin-marked brains only in the D3 developmental group at D7E, but in all developmental groups at D12E. GFAP-marked brains showed significantly increased brain-to-head width ratios only in the D5 group at D7E and in the D1 and D3 groups at D12E. DZN-exposed planarians showed significantly increased brain-to-head width ratios in synapsin-marked brains in the D1 and D5, but not D3, developmental groups at D12E, and in GFAP-marked brains in the D5 group at D12E.

MT and PFS caused both significant decreases and increases in relative brain size that were dependent on the timing and duration of exposure. Following MT exposure, significantly smaller brain sizes were only seen in GFAP-labeled brains at D7R/D7E in the D1 developmental group, but not in the later developmental groups. In contrast, at D12E, the D3 developmental group showed statistically significant increased relative brain size, while no other effects were seen in the other developmental groups. PFS also caused decreased relative brain sizes in the D1 developmental group at D7R/D7E using both synapsin and GFAP. Significantly reduced relative brain sizes were also observed in the D3 group at D7E in GFAP-labeled brains and in the D5 group at D7R in synapsin-labeled brains. In contrast, the D3 and D5 developmental groups had increased relative brain sizes in synapsin-labeled neurons at D12E.

**Fig. 5:**
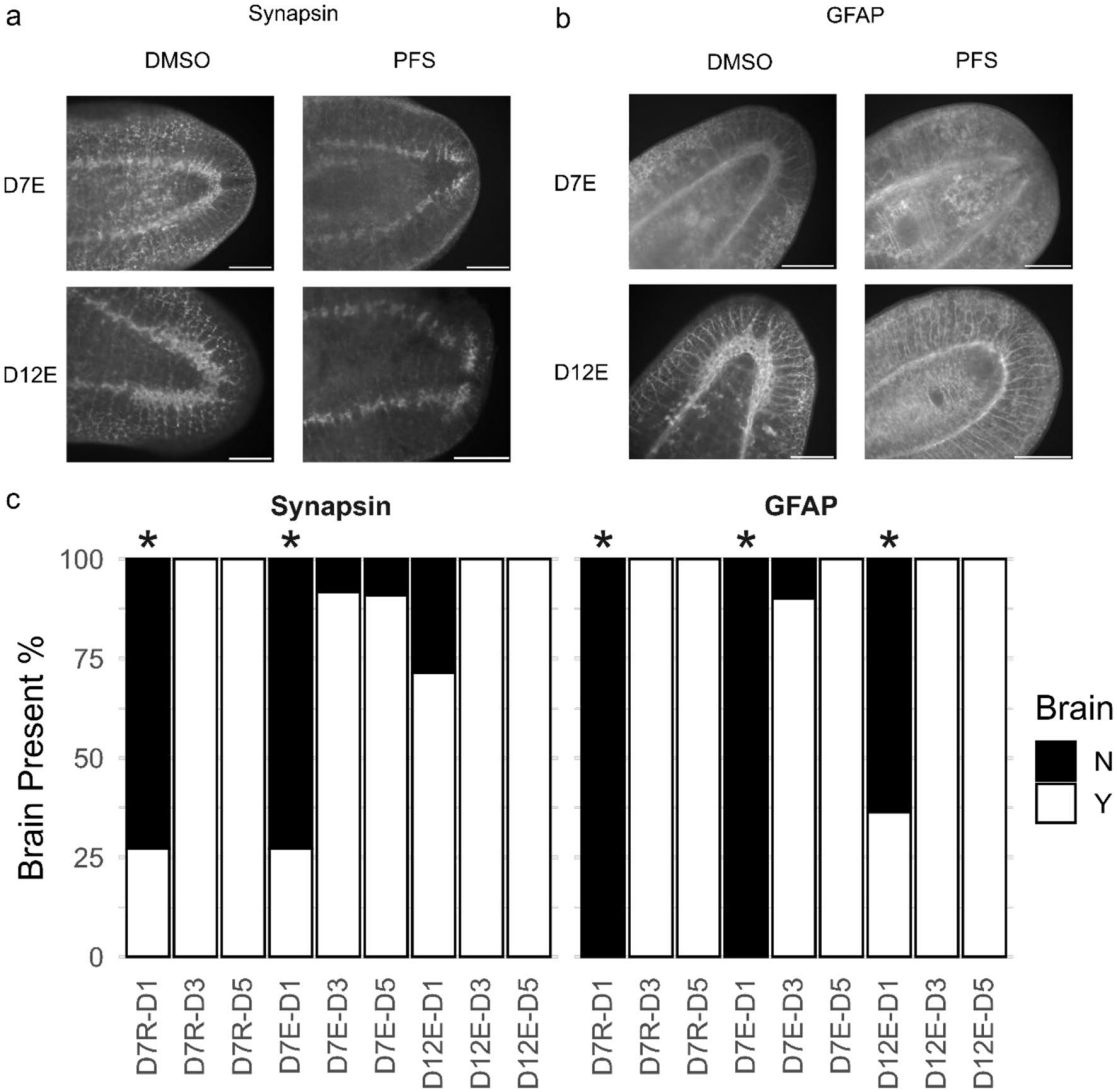
Percentage of PFS exposed worms that failed to regrow brains. (**a**, **b**) Representative images of 0.5% (v/v) DMSO control planarians with fully formed brains or PFS-exposed planarians with incomplete brain regeneration, defined as either failure to reconnect across the commissure or lacking visible neuropil (see Supplementary Fig. 1) using antibodies against (**a**) synapsin or (**b**) GFAP. (**c**) Percentages of planarians with brains present (Y, white) or absent (N, black) within a single developmental group and measurement time point. For the D1 group, D7R and D7E are the same timepoint; therefore, this point was reproduced for comparison. Significant differences compared to the time-matched DMSO controls (not shown, all contained developed brains) across developmental groups at the same time point are marked with a * where *p* < 0.05 using a Fisher’s exact test. N = 5–13 planarians per developmental group, screening time, and antibody

Strikingly, we observed that some PFS (and in some rare cases MT) exposed planarians did not fully develop a brain (Fig. 5). These cases were labeled as having relative brain sizes of 0, leading to the large distribution of values seen in some conditions (Fig. 4). The incidence of lack of brain formation in PFS-exposed planarians was significantly different from controls in the D1 developmental group at all time points across both antibodies, except for D12E with synapsin-labeling, and was not significant when exposure began later during development (Fig. 5c).

Finally, we wanted to determine if phenotypic differences observed between developmental groups might be due to differential AChE inhibition; thus, we measured AChE activity at D12E using an Ellman biochemical assay in the earliest and latest developmental groups (D1 and D5). There were no significant differences in AChE inhibition between planarians in groups D1 or D5 (Supplementary Fig. 2). This suggests that the observed differences between developmental groups are likely not due to differences in AChE inhibition.

## Discussion

### Some behavioral outcomes depend on the developmental stage at OP exposure

This study aimed to identify potential windows of vulnerability to ascertain which neurodevelopmental key events are vulnerable to exposure to 5 selected OPs (CPF, DDVP, DZN, MT, PFS). Our screening results, particularly those of the D1 developmental group, corroborate previous findings that these 5 OPs induce different phenotypes (Ireland et al. 2022b). Here, we also found that the impact of developmental stage during exposure differed across the OPs and across specific readouts.

For example, in DDVP-exposed planarians, speed and resting in the dark and locomotor bursts at D12E only showed statistically significant effects in the D3/D4 or later development groups, respectively. These trends could indicate DDVP acts on a target only found at later stages of planarian development, leading to a later window of vulnerability. Chronic exposure to a non-systemically toxic dose of DDVP in adult male rats was found to induce oxidative stress, mitochondrial abnormalities, and degeneration of dopaminergic neurons, concomitant with behavioral defects including reduced locomotion and motor impairment (Binukumar et al. 2010). In planarians, differentiation of specific neuronal subtypes begins around 36 h post regeneration (Ross et al. 2017) and dopaminergic neurons are first visible in regenerating *D. japonica* beginning on the 3rd day of regeneration, with full recreation of the dopaminergic neuronal network occurring during days 5–7 (Nishimura et al. 2007). Thus, it is possible that the increased sensitivity of more developed planarians reflects effects on specific neuronal cell types, such as dopaminergic neurons, that are not present at earlier stages.

These observations beg the question of why these behavioral effects were not also seen in the earlier developmental groups given that DDVP exposure included days 3–5 of regeneration in all developmental groups. One possibility is that due to the static exposure conditions, DDVP concentrations on days 3–5 were lower in developmental groups D1 and D2, due to OP adsorption into the plastic, hydrolysis (Di Consiglio et al. 2020), and/or detoxification (Alejo-González et al. 2018). This reduced concentration may have been below the threshold to cause significant toxicity in all the same endpoints. Some behavioral defects, such as reduced speed and resting in the dark, were still observed in the D1 and D2 developmental groups. Another possibility is that earlier exposure to DDVP set the regenerating planarians on a different developmental trajectory than when exposure began later, and thus different types of phenotypes were affected in earlier vs later exposures.

PFS also demonstrated increased potency when exposure began later during development as significantly increased lethality was only observed in the D5 developmental group. Previously, we showed that regenerating worms exposed to 10 µM PFS (the same concentration used here) at D1 did not experience significant lethality, but adult planarians did (Ireland et al. 2022b). Adult planarians have been found to be more susceptible to lethality induced by a variety of chemicals compared to regenerating planarians (Zhang et al. 2019; Ireland et al. 2022b, 2025b; Bayingana et al. 2023). While this initially appears to contrast with mammalian studies in which developing organisms are considered generally more vulnerable to toxicant exposure (National Research Council (U.S.) 1993), lethality at equivalent nominal concentrations is not a direct measure of developmental sensitivity. In mammals, placental buffering, evolving metabolic capacity, and dynamic developmental processes can decouple exposure, lethality, and long-term neurodevelopmental outcomes (Scheuplein et al. 2002). In planarians, regenerating and intact worms are similar in size and xenobiotic metabolic capability (Ireland et al. 2022a), but differ in physiological state, basal metabolic activity (Osuma et al. 2018), and potentially toxicokinetics, which can influence systemic toxicity. Consequently, higher lethality in adult planarians may reflect increased whole-organism toxicity instead of greater neurodevelopmental vulnerability. Thus, the observed PFS effects may be due to enhanced systemic toxicity in more developed planarians. This distinction emphasizes the importance of evaluating functional and behavioral endpoints in both regenerating and adult animals to differentiate developmental neurotoxicity from general systemic toxicity.

For some OPs, no clear patterns of differential effects due to exposure timing could be observed across any readout. For example, most of the behavioral effects observed following CPF exposure were not affected by the timing of exposure. Similar results have been found in rats, as exposure to CPF during early or late gestation resulted in similar behavioral effects measured during adolescence and adulthood, particularly attenuating the effect of the muscarinic receptor antagonist scopolamine on working memory (Icenogle et al. 2004). The authors propose these findings suggest a wide window of vulnerability to CPF, wherein different initial molecular initiating events during the different developmental stages eventually converge to the same adverse outcome (effects on cholinergic hippocampal function) (Icenogle et al. 2004).

In contrast, results from developing zebrafish found that exposure to CPF later during development caused greater behavioral effects than earlier exposures, though exposure duration and developmental state at evaluation varied across groups (Richendrfer and Creton 2015). In humans, early developmental events may be more susceptible to OP exposure, particularly for CPF, as systematic reviews of epidemiological studies have found that across studies more consistent statistical associations with negative effects on neurodevelopment were found for prenatal OP exposure than with postnatal exposure, where few statistically significant effects have been reported, even when evaluating the same cohort (González-Alzaga et al. 2014; Sapbamrer and Hongsibsong 2019).

## Shared behavioral effects independent of exposure timing may be due to AChE inhibition

All tested OPs, besides DDVP, had effects on speed in the light period at D7R or D7E irrespective of the developmental day that exposure began. This trend was mostly intact at D12E as well, except for DZN that only caused effects if exposure began in D3 or later. Similarly, increased stickiness, which we have previously shown to be correlated with increased cholinergic toxicity (Hagstrom et al. 2018; Ireland et al. 2022b), was observed at D7E/D7R in all developmental groups exposed to CPF, DDVP and DZN. MT had less conserved effects on stickiness at D7E (only in D2 and D4) but showed more robust results at D12E as effects were observed in D1-D4. The decreased effectiveness of MT to induce increased stickiness on D7E may be due to its weaker inhibition of *D. japonica* AChE. PFS also had relatively few effects on stickiness, which may be due to the relatively high toxicity observed at the tested concentration as we have previously seen that systemic toxicity and lethality can cause decreased stickiness (Ireland et al. 2022b). We have previously shown that both speed in the light and stickiness were often affected by OP exposure and were also impaired in regenerating planarians exposed to the nicotinic acetylcholine receptor agonist anatoxin-A (Ireland et al. 2022b). Together, these findings suggest that these shared effects on speed in the light and stickiness may be due to cholinergic toxicity from the shared effects of these OPs on AChE activity.

Given the observations that several cholinergic-related endpoints were affected independent of exposure timing, we hypothesized that AChE inhibition would be similar between early and late exposure groups, when measured on day 12 of exposure. We therefore measured AChE inhibition on day 12 for the D1 and the D5 groups. At the concentrations tested here, all the tested OPs induced considerable inhibition of AChE activity (at least ~ 70% inhibition) following 12 days of exposure and this was not affected by exposure timing. This result supports the observation that cholinergic-related endpoints were not affected by exposure timing and also suggests that AChE inhibition levels alone, as measured on day 12, cannot explain the observed OP- and developmental stage-dependent differences. Differences in AChE inhibition may have been present at earlier exposure time points reflecting developmental differences in toxicokinetics or toxicodynamics but inhibition eventually reached the same steady state seen at D12E. Such differences could contribute to phenotypic differences between developmental groups, in addition to possible effects of the OPs on other targets.

### Effects on brain size differ by OP and for some OPs by developmental timepoint

We previously found that 50 µM CPF reduced the brain-to-head width ratio in regenerating planarians after 15 days of exposure (Hagstrom et al. 2015), while exposure to DDVP up to 0.5 µM had no effect. Thus, we predicted that different OPs would cause different effects on planarian brain morphology and wanted to further test if changes in brain size would be dependent on the developmental stage at which the OPs were applied and whether these could explain some of the observed behavioral defects. First, we focus on effects on neurons, as visualized using an anti-synapsin antibody, as this is a common tool for evaluating planarian neuronal morphology (Hagstrom et al. 2015; Majid et al. 2022).

PFS caused the greatest effects on planarian brain morphology with some PFS-exposed brains failing to develop completely. This effect was most pronounced at D1, suggesting a specific window of vulnerability within the first 48 h of development (time between start of exposure in D1 and D3). Some of these brains failed to develop neuropil at all while others developed neuropil but the brain did not reconnect across the anterior commissure. Reconnection across the axonal commissure normally occurs at 3 days of development/regeneration (Ross et al. 2017), thus it makes sense that exposure after this stage, when reconnection is already complete, would not lead to the same level of defect. Failure to develop the neuropil appropriately could be due to effects on cell proliferation or migration, which occur during the first 3 days of planarian neural regeneration (See Fig. 1; Cebrià 2007; Ross et al. 2017)). Studies in cultured human peripheral blood lymphocytes have found that PFS is cytotoxic and at sublethal concentrations is also genotoxic, potentially explaining its potential effects on planarian cell proliferation (Prabhavathy Das et al. 2006). Notably, PFS is a chiral compound and the two enantiomers have been shown to display different toxicities. For example, the (-) enantiomer induced greater reactive oxygen species and cell death than the ( +) enantiomer and the racemic mixture (Lu and Yu 2014) in rat PC12 cells. Here, we used the racemic mixture of PFS and thus cannot parse out enantiomer-specific effects.

PFS only showed significant effects on reduced brain size at D7R/D7E but not at D12E, suggesting the effects likely represent delays in development, rather than failure to develop. However, PFS produced interesting and variable results at D12E across the different developmental groups. Although normalized brain sizes were not significantly different in the D1 developmental group at D12E, there appeared to be a bimodal distribution of effects, where some planarians failed to develop brains (although this was not significantly different from controls when evaluating neurons), but the ones that did develop had normal sizes. Interestingly, the later developmental groups showed the opposite results as increases in relative brain size were observed in the D3 and D5 developmental groups at D12E. This may also correlate with the observation that significant lethality was only observed in the D5 development group. Thus, taken together, there appears to be different mechanisms involved depending on when PFS exposure begins, which warrants further investigation.

The other OPs showed weaker effects on neuronal morphology. CPF, DDVP, and DZN exposure led to significantly increased relative brain size, primarily at D12E, whereas MT had no effect on neurons. CPF showed a potential early window of vulnerability as effects were only seen in the D1 developmental group, whereas DDVP had similar effects in all developmental groups and DZN had effects in the D1 and D5 groups. Of note, our previous study found decreased relative brain size in regenerating planarians after 15 days of exposure/regeneration to CPF (Hagstrom et al. 2015). The previous study used a higher CPF concentration of 50 µM compared to 17.8 µM used here, suggesting different mechanisms at the lower and higher concentrations.

### Different OPs affect developing neurons and glia differently

We also evaluated effects on glia using an antibody against GFAP (Ireland and Collins 2023). Evaluating both neurons and glia provides complementary insight into the mechanisms of OP developmental neurotoxicity, especially as neurons and glia develop on different timelines, with neurons first appearing at 3 days post regeneration and glia appearing between 5 and 6 days (Chandra et al. 2023). Some OPs, such as PFS and DDVP, showed similar trends in effects in both neurons and glia, whereas some OPs—namely CPF and MT—only showed effects in one cell type and not the other. While CPF increased relative neuronal brain size in the D1 developmental group at D12E, no significant effects were observed in glial brain morphology. It has been shown that CPF inhibits DNA synthesis to a greater extent in vitro in rat gliotypic C6 cells than in neurotypic PC12 cells (Qiao et al. 2001), suggesting greater sensitivity of glial cells to CPF exposure. However, the opposite result was found in aggregating cell cultures of fetal rat telencephalon containing mixed cultures of both neurons and glial, where CPF only caused a slight increase in GFAP-staining, but at concentrations above which caused decreases in neuronal activity (Zurich et al. 2004), thus demonstrating greater sensitivity in neurons, similar to our results in planarians. In rats, in vivo effects on GFAP-expression were only observed following late postnatal exposure (PN 11–14), and not prenatal exposure, with the nature of effects dependent on the time of evaluation as GFAP levels were first lower than controls, then rebounded to normal levels and subsequently were upregulated relative to controls (Garcia et al. 2002). Thus, it is possible that the exposure and evaluation paradigm used for planarians here could not capture similar dynamic changes. Moreover, our measures of relative brain size quantified gross changes in overall morphology/size but do not quantify expression levels and thus are likely not sensitive enough to detect these types of changes.

In contrast, MT only showed effects in GFAP-labeled brains and specifically in the D1 developmental group at D7R/E, indicating a potential window of vulnerability during the first 48 h of planarian neurodevelopment. This timing is interesting given that glia first appear within the regenerated planarian brain around days 5–6. These results could indicate disruption of earlier processes, such as progenitor proliferation or migration, that have downstream effects on glia development. Of note, lower relative brain sizes were also seen in neurons but the results were not significantly different from controls. Decreases in brain size have also been observed in zebrafish larvae exposed to MT (Richendrfer and Creton 2015). Moreover, malathion was found to induce cytotoxicity in human and rat astrocyte cell cultures (Hsu et al. 2019). In our study, the effects of MT on relative brain size were not seen at D12E, similar to PFS, suggesting potential delays rather than complete disruption of glia development. These transient effects may also reflect reversible changes that were repaired by the end of the 12 day exposure.

Interestingly, as mentioned above, we observed that exposure to CPF, DDVP, and DZN caused brains to have larger size ratios than their associated controls. One possible explanation for this phenotype could be increased inflammation following OP exposure. Inflammation, caused by activation of astrocytes, has been observed after OP exposure in in vitro and mammalian in vivo models (Chen et al. 2024). Notably, although planarians have glial-like cells, primarily with astrocyte-like identity, they only have an innate immune response (Kangale et al. 2021) and no microglia (Gonzalez et al. 2025); thus, it is unclear whether neuroinflammation would be activated in the same way in this organism and requires further investigation. DDVP and DZN increased relative brain size in both neurons and glia, which may relate to the interrelationship of these two cell types, as neurons are required for proper glial development and glia are thought to support and regulate neuronal morphology (reviewed in (Chandra et al. 2023)). In contrast, we found that CPF increased relative neuronal brain size only. It is possible that this increase in brain size may be due to other mechanisms besides inflammation, such as disrupted axonal guidance or increased proliferation. To distinguish between changes in neuronal processes versus cell bodies, future studies could compare anti-synapsin IHC with DAPI labeling. Alternatively, the apparent increase in brain size may reflect a relative change caused by loss or reduction of non-neural tissues rather than a nervous system–specific effect. One way to test for specificity to the nervous system would be to label neuronal subpopulations (Ross et al. 2015) to determine whether particular neuronal lineages are selectively affected.

### Limitations and opportunities

The work described here provides initial insights into potential windows of vulnerability for 5 OPs (CPF, DDVP, DZN, MT, and PFS) and provides a framework for studying OP-specific developmental sensitivity using planarians. It also highlights important considerations and limitations for interpreting and extending this work. Regarding the behavioral assay, caution should be taken to not overinterpret hits without future confirmation. Some behavioral readouts showed significant effects in a single developmental group without a clear temporal pattern, such as the phototaxis effects observed with DDVP, DZN, or MT. While these may be due to specific vulnerability at these developmental timepoints, they could also represent statistical false positives. Organismal behavior is intrinsically noisy with many factors, such as diet, chemical stocks, and individual organism differences, contributing to variability (Würbel 2000; Richter et al. 2009). For planarian behavioral screening, we have observed that while determination of potency is generally conserved when looking across endpoints, exact reproduction of hits in specific behavioral readouts across independent experiments can be challenging (Pacis et al. 2025). To reduce variability in this study, the same chemical stock was used for all developmental groups and all groups were exposed and screened together. Another, important future step to reduce variability in planarian behavioral screening will be to assess the robustness and information content of individual endpoints to define a minimal set of reliable, informative measures and to define “behavioral modules” that contain correlated behaviors that would be expected to change together, thus helping to identify false positives. Using data-driven criteria to select a minimal set of endpoints would be expected to improve consistency across experiments, facilitating interpretation and, where relevant, cross-laboratory comparisons.

For feasibility, IHC experiments were only performed on developmental groups D1, D3, and D5. To solidify the observations presented here and refine the windows of vulnerability, it would be interesting to evaluate the intermediate developmental windows, especially since we observed some effects that were dependent on exposure timing. Using synapsin and GFAP antibodies and whole brain fluorescent imaging, we were only able to identify gross morphological changes. Future work could expand these studies using neuronal subtype specific antibodies (Ross et al. 2015) to test how specific cell types are affected and whether relative changes in brain size are due to changes to the brain or to the surrounding tissue. Integrating these cellular analyses with transcriptomics would allow earlier molecular changes to be identified that may precede and drive the structural alterations detected by immunostaining. By studying multiple developmental time points using transcriptomics, it will be possible to pinpoint molecular targets and key events that differ across OPs and developmental stages, providing a more mechanistic understanding of how exposures translate from molecular perturbations to tissue-level outcomes.

Some effects on both behavior and neuroanatomy were dependent on the developmental stage during which exposure occurred. Interpretation of these potential windows of vulnerability should also consider that there may be age-related changes in toxicokinetics. For example, increased mucus secretion following transection may impede chemical uptake early during regeneration, leading to greater toxicity when exposure begins later in development (less mucus). Future studies should dissect these potential toxicokinetic differences and how they may relate to phenotypic differences. This could be achieved by measuring internal OP concentrations with exposure beginning at different stages of development and following different lengths of exposure to understand the dynamics of OP uptake. Furthermore, while we have shown that *D. japonica* can bioactivate CPF and DZN at all life stages and contain important OP detoxification enzymes (carboxylesterase and paraoxonase) (Ireland et al. 2022a), future experiments should better characterize these activities, including their kinetic relationships (i.e., balance of bioactivation vs detoxification) and the genes/proteins responsible for their action. By integrating these kinetic and enzymatic analyses with phenotypic observations, future research can establish a mechanistic framework connecting OP exposure, bioactivation, detoxification, and neurodevelopmental outcomes.

## Conclusion

In this study, we have shown that *D. japonica* can be used to compare outcomes after exposure to OPs during different developmental stages to identify windows of vulnerability to OP exposure during neurodevelopment. The identified windows, behaviors, and morphological endpoints can be used as a basis for future mechanistic research to identify the key events and molecular targets of these OPs.

## Supplementary Information

Below is the link to the electronic supplementary material.

Supplementary file1 (XLSX 170 KB)

Supplementary file2 (XLSX 70 KB)

Supplementary file3 (PDF 275 KB)

## Data Availability

All original data presented in this study are included in the article and Suppementary Material. Further inquiries can be directed to the corresponding author.
