# Supplementary material for: Timing of exposure impacts how organophosphorus pesticides affect the developing brain in the planarian Dugesia japonica
Source: Arch Toxicol. 2026 May 13;100(8):3719–36. doi: 10.1007/s00204-026-04415-x (PMC13379426; doi:10.1007/s00204-026-04415-x)
Supplement: Supplementary file 3 — Supplementary file3 (PDF 275 KB) [file 204_2026_4415_MOESM3_ESM.pdf]

Supplementary Figures

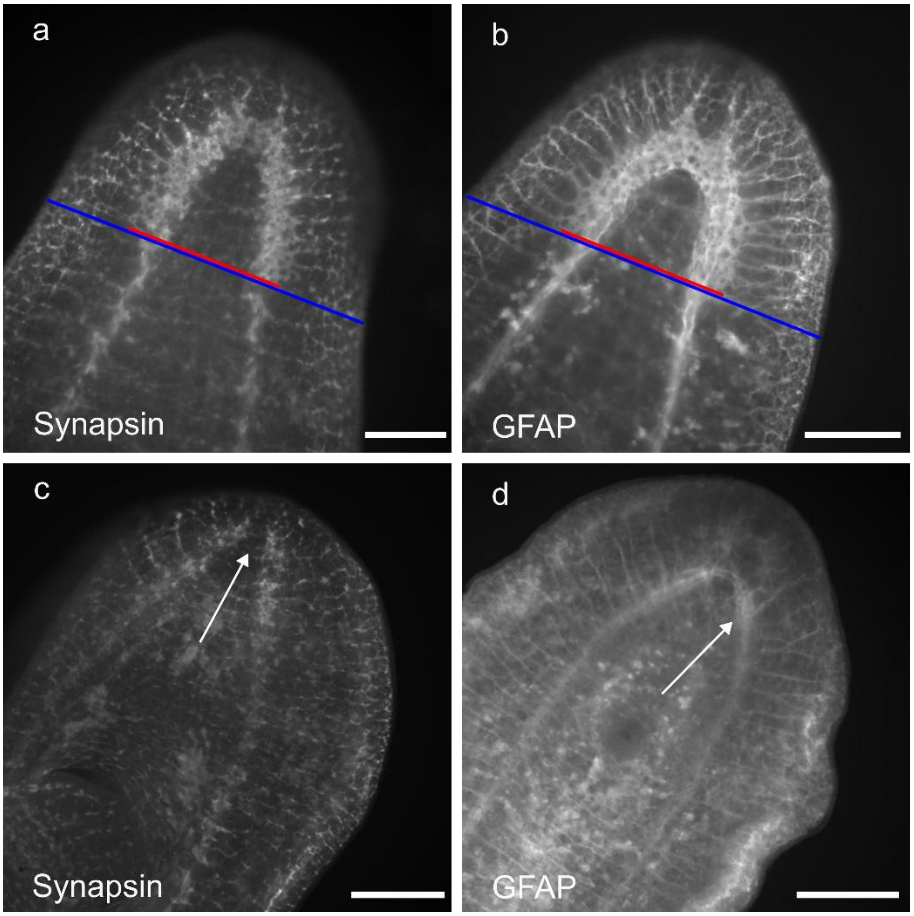

Supplementary Fig. 1 Measurement of relative brain sizes and examples of planarians with no brains. (a,b) Depictions of method of measurement of brain width (red line) and head width (blue line) in planarians labeled with antibodies against (a) synapsin or (b) GFAP. Representative images from 0.5% DMSO controls at D12E from the D1 group. (c, d) Examples of planarians with no brains in planarians labeled with antibodies against (c) synapsin or (d) GFAP. Representative images of profenofos-exposed D1 planarians on D7E. Arrows indicate conditions where a brain was incompletely formed with an example of failure of reconnecting across the commissure (arrow, c) or lack of visible neuropil (arrow, d). Scale bars are 200 µm.

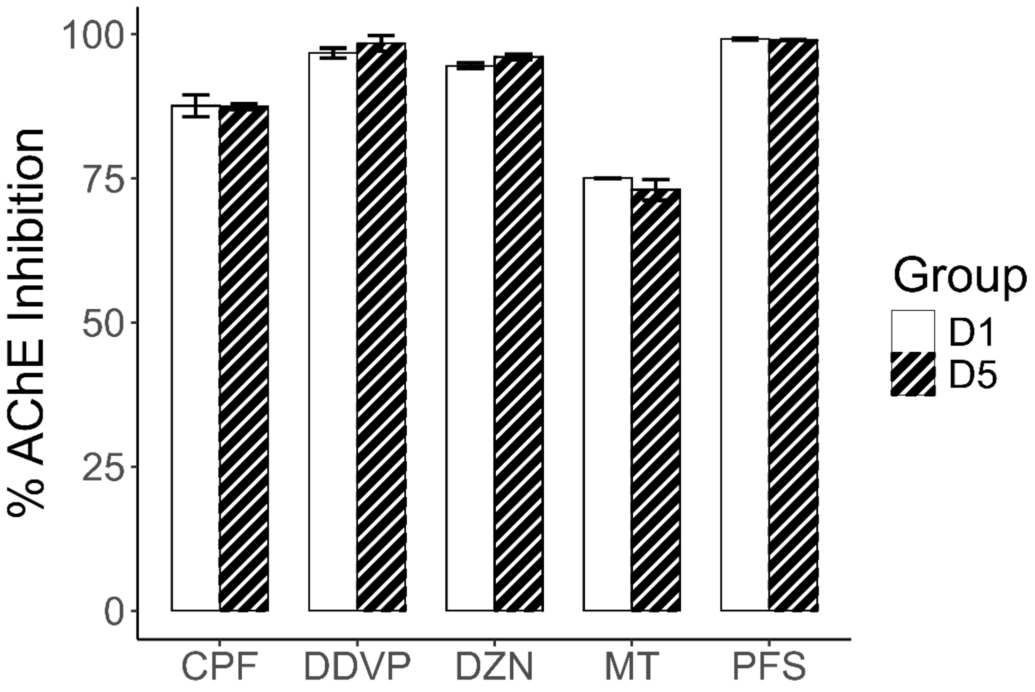

Supplementary Fig. 2 Acetylcholinesterase inhibition is not significantly different between D1 and D5 groups. Ellman assays were performed to measure acetylcholinesterase (AChE) activity at 12 days after OP exposure, and percent inhibition was compared to developmentally matched 0.5% DMSO exposed controls within the assay. Error bars are standard deviations from 2-3 independent experiments of N = 24 planarians per chemical per experiment. A 2-way ANOVA was performed in R comparing amputation date. P-values < 0.05 were considered significant. No significant differences were found.

Supplementary Tables

Supplementary Table 1: Definitions of neurodevelopmental key events in Figure 1

**Key Event**	**Description**
Proliferation	Precursor cell proliferation.
Migration	Movement of precursor cells to final locations.
Neurogenesis	Neuronal cell differentiation.
Gliogenesis	Glial cell differentiation, not including maturation time which can continue to full adulthood.
Neurite Outgrowth	Establishing dendritic and axonal projections.
Synaptogenesis	The formation of synapses between neurons.
Network Formation	Begins with neurite outgrowth, continues through synaptogenesis to form connections, then myelination and synaptic pruning to form the mature network.

Supplementary File Legends

Supplementary File 1 Mass spectroscopy analysis of chemical purity. Zipped file containing purity analysis reports from Lotus Separations.

Supplementary File 2 Compiled data and statistical analysis for planarian behavioral data. Excel file contains the compiled values generated from MATLAB analysis of planarian behaviors, the p-values generated by statsitsical analysis in R, the # of planarians used in determining the raw compiled behavior values, and the behavior values averaged for all worms in an individual plate (Runs A, B, and C) for each endpoint (separate sheet). Dichlorvos (DDVP) was run separately than other OPs and is listed next to the DMSO control values that were run at the same time. Each sheet also contains the values for the additional DMSO controls that were used to produce the biological relevancy cutoff values. The final sheet contains the biological relevancy cutoff values of the 5th and 95th percentiles for each behavior and group. Significant values of the compiled behavior data as determined by $p < 0.05$ are in bold, red text.

Supplementary File 3 IHC data. Excel sheet consisting of : 1) "ByWormAverage" containing the brain-width-to-head-width ratio value as averaged between multiple experimenters (Average_Value) and the standard deviation of these calculations (StdDev_Value). N_count is the number of calculations that were used to generate each Average_Value. Worm Number is the number of the worm in each group. Values that contain no Average_Value and no N_count had issues with worm placement or staining quality and were excluded from final analysis. 2) "GroupAverage" contains the median brain-width-to-head-width ratio for each group (median_value) and the number of planarians used to generate this value (N_Count). Medians were used as this data was not normally distributed. 3) "KruskalWallisResults" contains the statistical results of the Kruskal Wallis test with post-hoc Dunn's test for the group comparisons between DMSO controls and each OP to determine effects on relative brain size. Significant values as determined by $p < 0.05$ are in bold red text. 4) "Fisher Test" contains the statistical results of the Fisher Exact test of the comparison of PFS exposed planarians to DMSO group matched controls to determine if failure to regenerate brain tissue is statistically significant across groups. N values listed are for the combination of animals in both the DMSO group and the Profenofos exposed groups. Significant values as determined by $p < 0.05$ are in bold red text.

Supplementary File 4 Supplementary figures, tables and file captions. This document.
